# Sika deer presence affects the host–parasite interface of a Japanese land leech

**DOI:** 10.1002/ece3.6344

**Published:** 2020-05-19

**Authors:** Kaori Morishima, Takafumi Nakano, Mineaki Aizawa

**Affiliations:** ^1^ United Graduate School of Agricultural Science Tokyo University of Agriculture and Technology Utsunomiya Japan; ^2^ Department of Zoology Graduate School of Science Kyoto University Kyoto Japan; ^3^ Department of Forest Science School of Agriculture Utsunomiya University Utsunomiya Japan

**Keywords:** Anura, blood meal, *Cervus nippon*, Haemadipsidae, host preference, iDNA, mitochondrial DNA

## Abstract

Since the 1990s, increasing populations of a blood feeding land leech (*Haemadipsa japonica*) have become a serious issue in several Japanese prefectures, and it may be caused by the increases in sika deer (*Cervus nippon*) populations seen over the last quarter of the century. Therefore, this study aimed to reveal the host animal species of *H*. *japonica* using iDNA (vertebrate DNA isolated from invertebrates) and to test the hypothesis that the increasingly widespread distribution of sika deer results in increased *H*. *japonica* populations through changes to the host–parasite interface. We amplified mitochondrial DNA 16S ribosome RNA fragments from iDNA isolated from the blood clots of *H*. *japonica* collected across Japan. We identified 17 host animal species, including four orders of Mammalia (Carnivora, Artiodactyla, Rodentia, and Lagomorpha) and two orders of Amphibia (Caudata and Anura). The sika deer was the dominant host species of *H*. *japonica*. Additionally, the host animal species composition of *H*. *japonica* differed according to the presence or absence of sika deer. In the sites where sika deer were not found, Anura (frog) species were the most commonly identified hosts of *H*. *japonica*. These results suggest that the increases in *H*. *japonica* populations might have occurred via a change in host preference to sika deer. This change might be driven by the increases in sika deer populations and subsequent increase in the frequency that *H*. *japonica* uses the sika deer as easy prey, as well as by sika deer providing more reproductive energy per blood meal than blood meal from frog species. The present study suggests that a more widespread distribution of sika deer resulted in an increase in *H*. *japonica* through a change in the host–parasite interface. Therefore, management that focuses on decreasing sika deer populations would likely be an effective method for the reduction of *H*. *japonica* populations.

## Graphical abstract

This paper revealed the host animal species of a blood land leech (*Haemadipsa japonica*) in Japan using vertebrate DNA isolated from blood feeding invertebrates (iDNA). Additionally, the host animal species of the leech differed according to the presence or absence of sika deer.

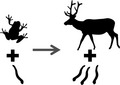

## 1 INTRODUCTION

Indirect samples from wild animals (e.g., scat, sound or sign surveys, and camera trapping) have been used to measure their biodiversity (Janečka et al., 2008; Pettorelli, Lobora, Msuha, Foley, & Durant, 2010; Sikes & Gannon, 2011). Though these monitoring tools are able to effectively detect mammals, detecting the biodiversity of small animals, including amphibians, is difficult. Recently, invertebrate‐derived DNA (iDNA), which is vertebrate DNA isolated from invertebrates (Schnell et al., 2015), has been isolated from several invertebrate species, such as blood feeding (sanguivorous) species (e.g., mosquitoes, midges, ticks, and terrestrial leeches), as well as carrion feeding (saprophagous) and dung feeding (coprophagous) species (Calvignac‐Spencer, Leendertz, Gilbert, & Schubert, 2013; Calvignac‐Spencer, Merkel, et al., 2013; Gariepy, Lindsay, Ogden, & Gregory, 2012; Gómez & Kolokotronis, 2017; Kent, 2009; Kocher et al., 2017; Lassen, Nielsen, & Kristensen, 2012). This iDNA has been used to investigate host–parasite interactions between invertebrates and their vertebrate hosts, to examine pathogen transmission via invertebrates, and as a vertebrate monitoring tool. iDNA is advantageous because it allows the collection of DNA from wild animals without direct contact, and it has high detection capability for multiple vertebrates because most inland blood‐sucking species have low host specificity (Calvignac‐Spencer, Leendertz, et al., 2013). Particularly, the feeding habitats of terrestrial leeches (Haemadipsidae) have been studied in a wide range of animal species (i.e., birds, mammals, and amphibians; Schnell et al., 2018). Thus, leech iDNA has been used to detect biodiversity and to create censuses of wild animals through identifying blood meals from a wide range of mammalian orders and by documenting their occurrence in target areas (Abrams et al., 2019; Schnell et al., 2012, 2015; Tessler et al., 2018), thereby contributing to the ecological and natural historical knowledge of various regions. Moreover, the iDNA from the blood of mosquitoes, flies, midges, and ticks has been used to identify the host species of target sanguivorous species, providing basic knowledge for infection control. However, few studies have been conducted into the applications of iDNA in forest ecology or wildlife management (e.g., Kent, 2009).

*Haemadipsa japonica* Whitman (Haemadipsidae) is a sanguivorous terrestrial leech species endemic to East Asia. The species has been documented in Honshu, Shikoku, Kyushu, and Yakushima islands in the Japanese archipelago (Borda & Siddall, 2010; Morishima & Aizawa, 2019; Nakano, 2017; Whitman, 1886). *H*. *japonica* was once restricted to mountainous regions (Aizawa & Morishima, 2018); however, since the 1990s, the distribution of *H*. *japonica* has expanded to areas influenced by human activities, including residential areas, which has become serious issues in several Japanese prefectures (Asada, Ochiai, & Yamanaka, 1995; Sugiyama & Sakaniwa, 2010). For example, the blood‐sucking damage caused by *H*. *japonica* causes mental stress to forest workers and increases the cost of administering pest‐control agents against this leech species (Morishima, Hayashi, & Aizawa, 2018).

In the last quarter of the century, the populations of several mammalian species, such as sika deer (*Cervus nippon*), Japanese serow (*Capricornis crispus*), and wild boar (*Sus scrofa*), have also increased (Natori & Porter, 2007; Takahashi, 2018; Takatsuki, 2009). In particular, since around the 1990s, the populations of sika deer have substantially increased; presently, the increased populations of sika deer strongly impact forest ecosystems and result in damages to agriculture and forestry (Noguchi, 2017; Takatsuki, 2009). These nearly concomitant increases in land leech and mammalian populations imply that the increases in *H. japonica* could be due to the increases in mammal populations, particularly sika deer (Sugiyama & Sakaniwa, 2010).

Previous studies have identified the host species of *H. japonica* with the aim of understanding the causes of *H*. *japonica* population increases using iDNA with polymerase chain reaction single‐stand conformation polymorphisms (PCR‐SSCP; Sasaki, Saito, & Harada, 2005; Sasaki & Tani, 2008; Kanagawa Prefecture, 2009), hybridization probes (Nakanojo Town, 2004), and immunohistological analyses (Yoshiba & Abe, 1989). However, results using these techniques are biased because, prior to the identification of host species, the length of the DNA fragment (i.e., electrophoresis patterns of DNA fragment bands) or the patterns of antigen–antibody reactions must be known for candidate host animals. Therefore if *H*. *japonica* feeds on a host animal that was not already identified as a candidate, the host would not be detected. iDNA identification based on nucleotide sequences such as current iDNA studies (e.g., Schnell et al., 2015; Schnell et al., 2012; Tessler et al., 2018) overcomes this bias because iDNA identification was conducted using vast nucleotide DNA database of multiple vertebrates. Therefore, host animal identification using nucleotide sequences of iDNA allows us to understand the causes of the current increases in *H*. *japonica* populations through the host–parasite interface of the land leech.

In this study, we aimed to reveal the host species of *H. japonica* in each site across Japan. We also aimed to test the hypothesis that the increasingly widespread distribution of sika deer resulted in the increases in *H*. *japonica* populations through a change in the host–parasite interface by comparing the host animals between sites where sika deer are present versus not present.

## 2 MATERIAL AND METHODS

### 2.1 Sample collection and DNA isolation

**FIGURE 1:**
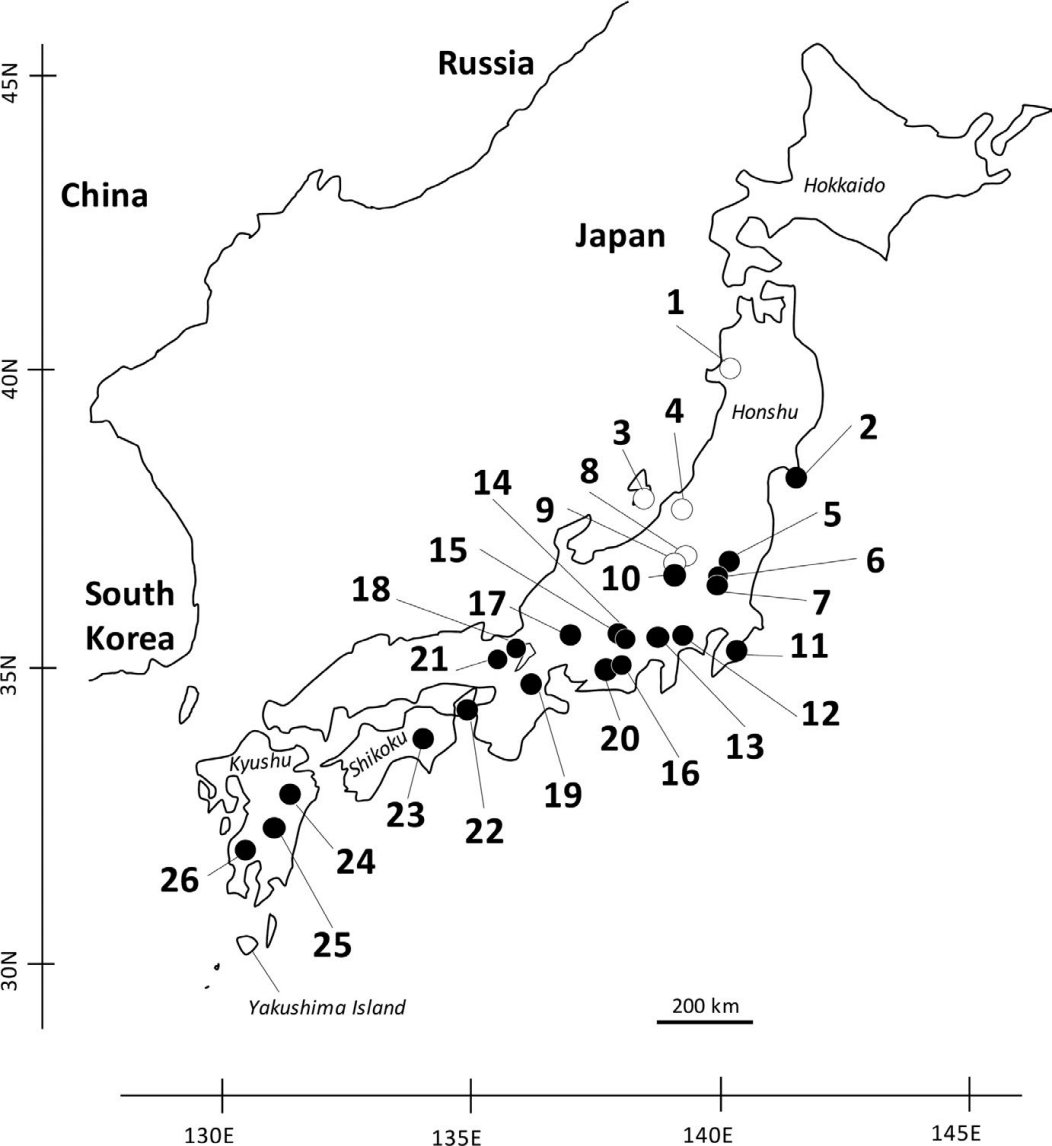
Collection sites of *Haemadipsa japonica*. Sites with solid and open circles indicate the presence and absence of the distribution of sika deer (*Cervus nippon*), respectively. Collection sites (1–26) correspond to these shown in Appendix S1

We collected 826 *H. japonica* samples from 26 sites, including five sites where sika deer are not present due to the site being situated on a solitary island (site no. 3) or in an area with deep snow in winter (site nos. 1, 4, 8, and 9) and 21 sites where sika deer are present (Figure 1; Appendix S1). The distribution of sika deer was determined based on a distribution map obtained from the Biodiversity Centre of Japan in the Ministry of Environment (http://www.biodic.go.jp/kiso/fnd_list.html). Land leech collection was performed by walking along the forest path to attract leeches and by gathering the leeches attached to the legs or other body parts of the researchers. The collected land leeches were stored in 99.9% ethanol until iDNA isolation. The mid‐bodies of the 826 collected leeches were dissected from the anterior to the posterior, and undigested blood clots in the digestive system were visually inspected (i.e., mainly crop and crop ceca). For the 216 leech samples with undigested blood clots, iDNA was isolated from the blood clots extracted from each sample (Appendix S1). Total genomic iDNA was isolated using a DNeasy Blood & Tissue Kit (Qiagen).

### 2.2 mtDNA sequencing

Mitochondrial DNA 16S ribosome RNA (rRNA) fragments were amplified from the iDNA isolated from 216 *H. japonica* individuals using the SCPH02500, 5ʹ‐TTACCAAAACATCACCTCT‐3ʹ and SCPL02981, 5ʹ‐ATCCAACATCGAGGTCGTAA‐3ʹ primers (Matsui, Rakotondraparany, Hasegawa, & Horai, 2007). Polymerase chain reactions (PCR) were performed in 15 µl reaction volumes, containing 10 ng genomic DNA, 1× PCR buffer, 0.2 mM of each dNTPs, 1.5 mM MgCl_2_, 0.2 µM of each primer, and 0.5 U GoTaq polymerase (Promega). PCR amplifications were performed as follows: an initial denaturation at 94°C for 10 min, followed by 45 cycles of 10 s at 94°C, 30 s at the annealing temperature of 51°C, 1 min at 72°C, and a final extension at 72°C for 10 min using a GeneAmp2720 PCR System (Applied Biosystems, PE Corp.). The PCR products were electrophoretically separated on a 2.0% agarose gel and visualized with ethidium bromide in 1× TAE; all products exhibiting a single DNA fragment were sequenced. Negative controls were used in PCR in our preliminary experiment, confirming no amplification. The products were purified using an ExoSAP‐IT (Affymetrix). Direct sequencing in both directions was conducted using the ABI PRISM BigDye Terminator version 3.1 Cycle Sequencing Kit (Applied Biosystems) on an ABI 3500 Genetic Analyzer. DNA sequences were subjected to visual inspection and were aligned using BIOEDIT 7.2.5.0 (Hall, 1999). The sequences obtained from leech iDNA were identified using the DNA Data Bank of Japan (DDBJ), Nucleotide BLAST Database, and blastn (http://blast.ddbj.nig.ac.jp/). A threshold of more than 98% identity was used. The leech iDNA that was identified as human was excluded from all analyses to remove the possibility of contamination through the experiment.

It was impossible to identify three salamander samples from site 15 to species level using the aforementioned primers. Therefore, we further sequenced mitochondrial cytochrome *b* (cyt *b*) fragments for the these three salamander samples using the following primers: L14010, 5ʹ‐TAHGGWGAHGGATTWGAWGCMACWGC‐3ʹ and H14778, 5ʹ‐AARTAYGGGTGRAADGRRAYTTTRTCT‐3ʹ, L14707, 5ʹ‐CAYTTYYTGYTMCCATTYYTAATTGCAGG‐3ʹ, and H15289, 5ʹ‐CTTCGGYTTACARGACCGATGYTTT‐3ʹ (Matsui, Nishikawa, Misawa, & Tanabe, 2007). PCRs were performed in 15 µl reaction volumes containing 20 ng genomic DNA, 1× PCR buffer, 0.2 mM of each dNTPs, 1.5 mM MgCl_2_, 0.5 µM of each primer, and 0.5 U GoTaq polymerase. PCR amplifications were performed as follows: an initial denaturation at 94°C for 5 min, followed by 35 cycles of 30 s at 94°C, 30 s at the annealing temperature of 55°C, 1 min at 72°C, and a final extension at 72°C for 5 min. We also sequenced PCR products by direct sequencing.

### 2.3 Data analyses

In the identification of host animal species, we treated *Rana tagoi* and *R*. *sakuraii* as a single species (called *Rana tagoi/sakuraii* hereafter) because they are not distinguished from each other by mitochondrial DNA because they showed nonmonophyly due to incomplete lineage sorting (Eto & Matsui, 2014). The differences in *H*. *japonica* host animal species composition among sites was examined using nonmetric multidimensional scaling (NMDS) and a Jaccard distance matrix based on the presence and absence of species between sites. A nonhierarchical clustering method, *k*‐means clustering, was performed to determine groups of sites. Indicator species analysis (Dufrêne & Legendre, 1997) was carried out to identify indicative species within the groups of sites determined by *k*‐means clustering. Multi‐response permutation procedures (MRPP) and analyses of similarities (ANOSIM) were used to test whether the host animal species composition differed according to sika deer presence. These analyses were performed using the “vegan” (Oksanen et al., 2017) and “labdsv” (Roberts, 2016) R packages in R 3. 6. 0 (R Core Team, 2019) for 14 sites of the 26 sites; 12 (sites 2, 8, 11, 13, 18–21, and 23–26) were excluded from these analyses due to a small sample size (*N* < 5; Appendix S1). MRPP and ANOSIM were performed between sites with sika deer (sites 5–7, 10, 12, 14–17, and 22) and sites without sika deer (sites 1, 3, 4, and 9).

This study examined the host–parasite interface and whether the host animal species of *H. japonica* differed according to sika deer distribution using generalized linear mixed models (GLMMs). In the GLMMs, the numbers of *H*. *japonica* individuals that fed on wild boar (*N*ss), Japanese serow (*N*cc), Carnivora (carnivore; *N*ca), and Anura (frog; *N*an) were used as response variables. The presence/absence of sika deer (0 = absence; 1 = presence; Dcn) and the altitude of each site (Alt) were used as explanatory variables and fixed effects. Sites identity was set as a random effect. The correlation between the two explanatory variables (Dcn and Alt) was assessed using a Spearman rank test in R. The details of the GLMMs model are shown in Table 1. The GLMMs were performed for 24 sites using the “glmmTMB” (Magnusson et al., 2020) package in R. Sites 17 and 18 were excluded from the GLMMs due to a lack of altitude information (Appendix S1). Predicted values and Wald 95% confidence interval (CI) were obtained using R.

**TABLE 1:** Result of generalized liner mixed models (GLMMs) used to assess the effects of altitude and presence of sika deer (*Cervus nippon*) on the number of each host animal species

Model	Response variable	Estimate (*SE*)	
		Alt	Dcn
Model 1 *N*ss ~ Alt + Dcn	*N*ss	–0.0010^NS^ (0.0019)	21.5361^NS^ (24,993.5067)
Model 2 *N*cc ~ Alt + Dcn	*N*cc	0.0027^NS^ (0.0018)	–1.6672^NS^ (0.9681)
Model 3 *N*ca ~ Alt + Dcn	*N*ca	–0.0002^NS^ (0.0012)	–1.3176* (0.6386)
Model 4 *N*an ~ Alt + Dcn	*N*an	0.0006^NS^ (0.0012)	–1.6524*** (0.4699)

Note

*N*ss, *N*cc, *N*ca, and *N*an indicate the number of *Haemadipsa japonica* samples that fed on wild boar (*Sus scrofa*), Japanese serow (*Capricornis crispus*), carnivores (Carnivora), and frog species (Anura) in the site, respectively. Alt and Dcn indicate altitude and the presence of sika deer in each site, respectively. Standard errors (*SE*) are shown in parentheses, NS, statistically non‐significant.

* *p* < .05.

*** *p* < .001.

## 3 RESULTS

### 3.1 Sequencing of mtDNA from iDNA of *H. japonica*

**FIGURE 2:**
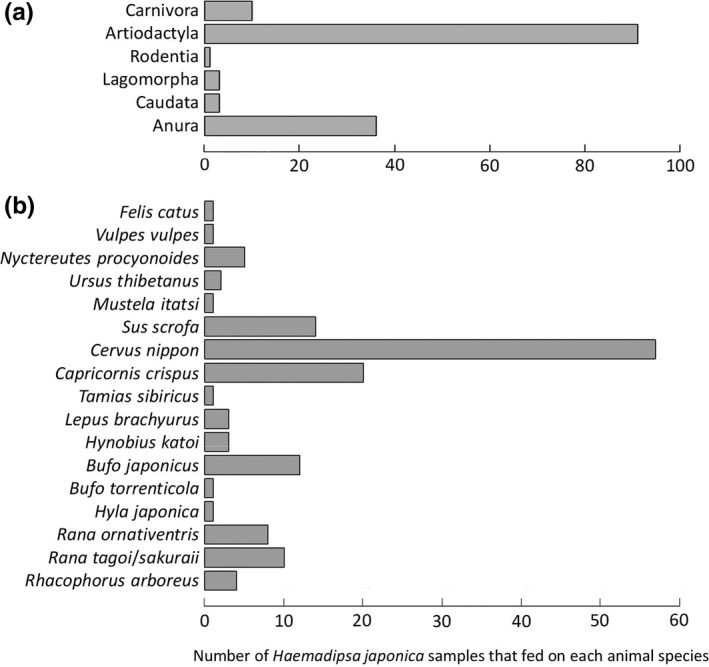
Total counts of different orders (a) and species (b) identified from *Haemadipsa japonica* invertebrate‐derived DNA (iDNA)

Of the 216 iDNA samples from the blood clots of *H. japonica* individuals, the host animal species could be identified in 144 iDNA samples, along with each unambiguous chromatogram of Sanger sequencing and mostly with more than 99% identity (Appendix S2). One host animal species was identified per iDNA sample from each individual leech. Total lengths of the sequenced mtDNA 16S rRNA and cytb fragments were 461–486 base pairs (bp) for 144 of the iDNA samples and 1,281 bp for the three salamander samples (Appendices S2 and S3). The host animal species of *H*. *japonica* included two different classes: Mammalia and Amphibia (Appendices S2 and S3). These consisted of four mammalian orders: Carnivora, Artiodactyla, Rodentia, and Lagomorpha, and two amphibian orders: Caudata and Anura. The orders that the leeches fed on most frequently were Artiodactyla (most frequent; the even‐toed ungulates) and Anura (second most frequent; frog) species (Figure 2). At the species level, 17 host animal species were identified. Of these, sika deer (*Cervus nippon*) was the dominant host species of *H*. *japonica* (Figure 2).

### 3.2 The differences in host animal species composition among sites

**FIGURE 3:**
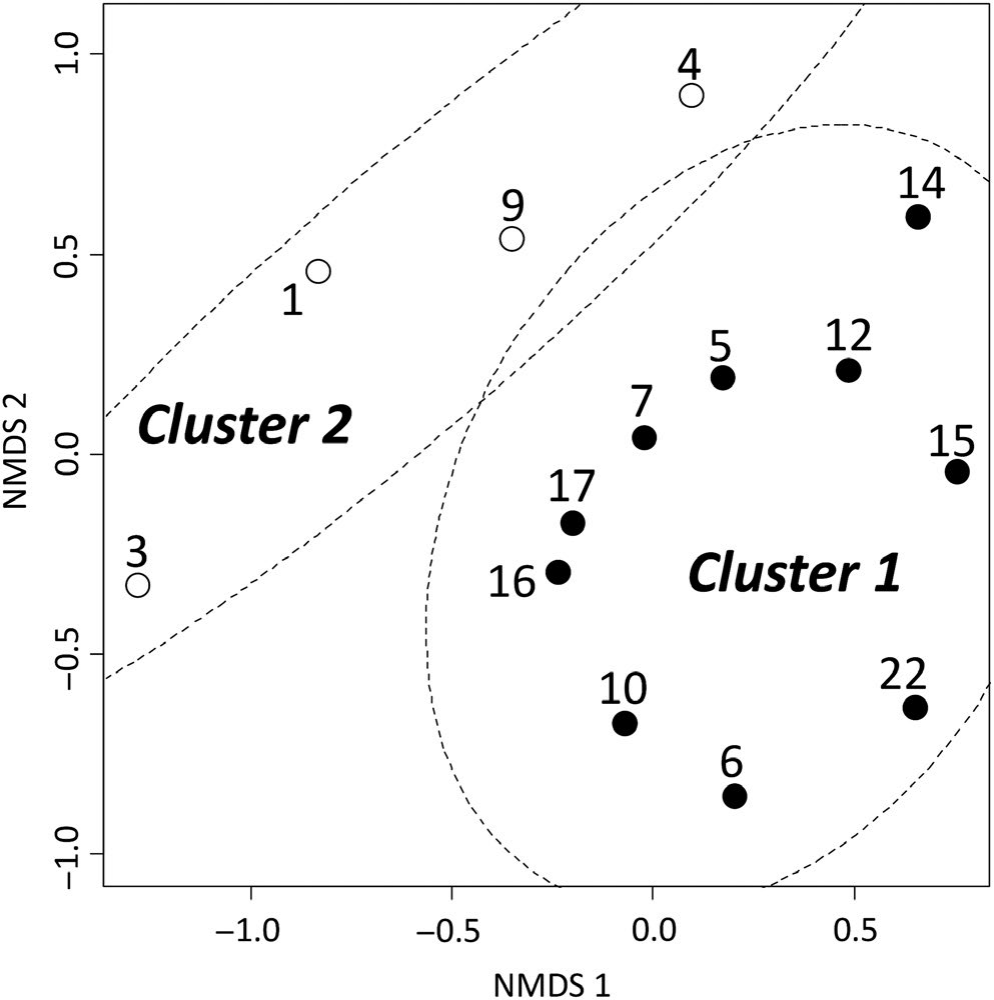
Result of nonmetric multidimensional scaling (NMDS) based on the presence and absence of host animal species among sites. Sites with solid and open circles indicate the presence and absence of the distribution of sika deer (*Cervus nippon*), respectively. Collection site numbers correspond to those shown in Appendix S1

The NMDS analysis and *k*‐means clustering produced two separate groups (clusters 1 and 2) of sites (Figure 3). Cluster 1 included 10 sites (sites 5–7, 10, 12, 14–17, and 22) in which sika deer are found. Cluster 2 included four sites (sites 1, 3, 4, and 9) in which sika deer are not found. The indicator analysis showed that the significant indicator species were sika deer (*Cervus nippon*; Indicator value = 0.900, *p* = .006; Table 2) in cluster 1 and the Japanese forest green tree frog (*Rhacophorus arboreus*; 0.750, *p* = .018; Table 2) in cluster 2. The stress value for the NMDS analysis was 0.15. The results of the MRPP and ANOSIM analyses indicated that there was a significant difference in host animal species composition between sites with sika deer (sites 5–7, 10, 12, 14–17, and 22) and sites without sika deer (sites 1, 3, 4, and 9; MRPP, *A* = 0.123, *p* = .002; ANOSIM, *R* = 0.714, *p* = .002).

**TABLE 2:** Results of indicator species analysis for host animal species identified from bloodmeals of *Haemadipsa japonica* collected from 14 sites

Host animal species	Cluster	Indicator value	*p*‐Value
*Cervus nippon*	1	0.900	.006
*Sus scrofa*	1	0.300	.518
*Hynobius katoi*	1	0.100	1.000
*Rhacophorus arboreus*	2	0.750	.018
*Nyctereutes procyonoides*	2	0.357	.531
*Capricornis crispus*	2	0.278	1.000
*Bufo japonicus*	2	0.278	1.000
*Rana tagoi*/*sakuraii*	2	0.278	1.000
*Felis catus*	2	0.250	.314
*Rana ornativentris*	2	0.250	.275
*Hyla japonica*	2	0.250	.273
*Mustela itatsi*	2	0.250	.269
*Ursus thibetanus*	2	0.179	1.000
*Lepus brachyurus*	2	0.139	1.000

### 3.3 Relationships among host animals

**FIGURE 4:**
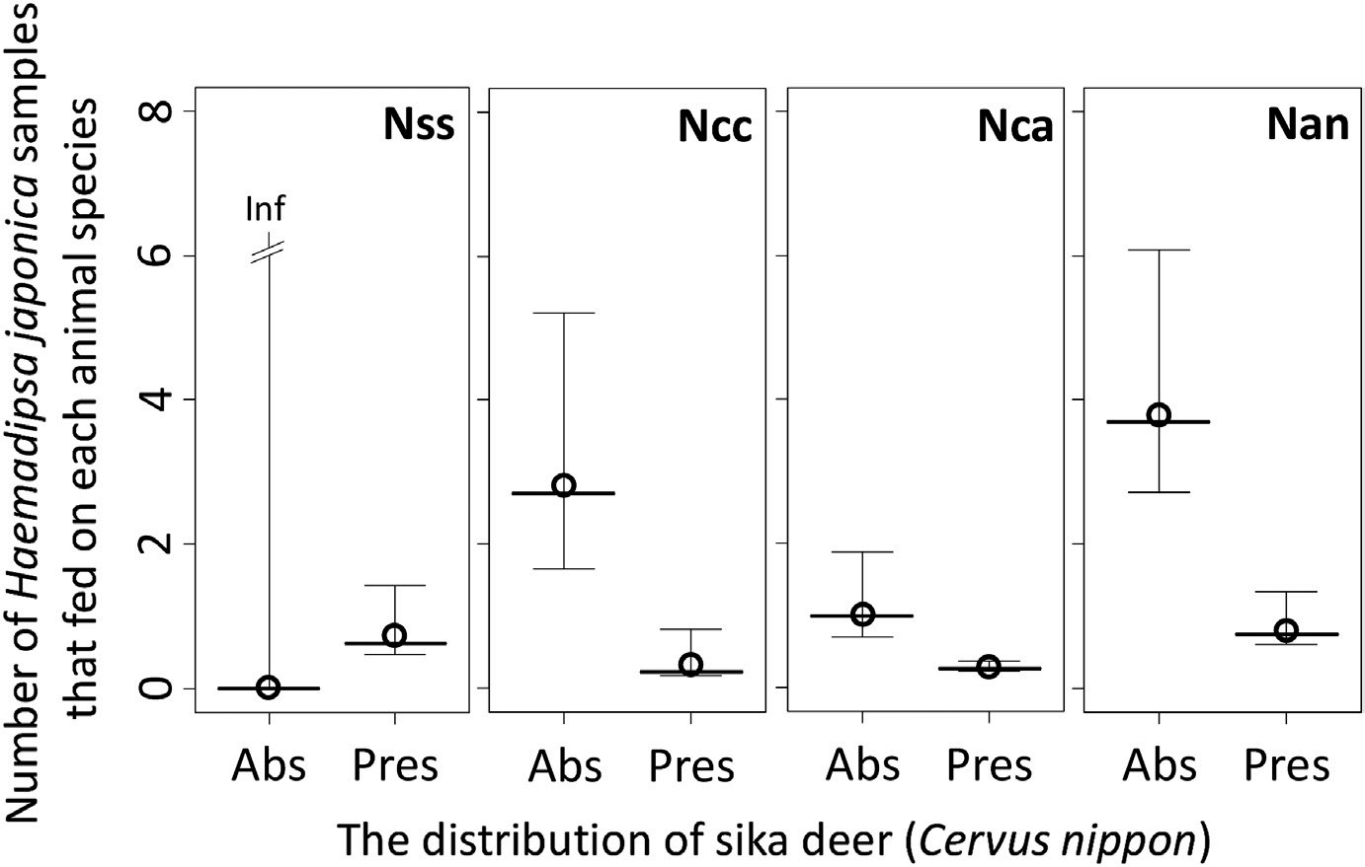
Predicted values and 95% confidence intervals obtained using generalized liner mixed models (GLMMs) to assess the effects of altitude and presence of sika deer (*Cervus nippon*) on the number of *Haemadipsa japonica* samples that fed on wild boar (*Sus scrofa*; *N*ss), Japanese serow (*Capricornis crispus*; *N*cc), carnivores (Carnivora; *N*ca), and frog species (Anura; *N*an) per site, which were shown by the presence (Pres) and absence (Abs) of the distribution of sika deer. Observed values per site for these numbers are also shown by open circles. Inf indicate that upper limit of 95% CI is infinite. Detailed data are shown in Appendix S4

The correlation between the presence of sika deer (Dcn) and altitude (Alt) was not significant, according to the Spearman rank test (*ρ* = 0.155, *p* = .467). The results of the GLMMs indicated that the presence of sika deer distribution (Dcn) significantly and negatively affected the number of *H*. *japonica* samples that fed on Carnivora (*N*ca) in Model 3 (*p* < .05) and on Anura (*N*an) in Model 4 (*p* < .001; Table 1). In the other models, estimates of the explanatory variables were not significant (*p* > .05). In each model, the predicted values mostly fit the observed values within 95% CI (Figure 4; Appendix S4).

## 4 DISCUSSION

### 4.1 The host animal species of *H. japonica*

The present study identified 17 host animal species of *H. japonica* (Figure 2; Appendix S2). Previous studies using genetic and immunohistological analyses revealed that the host animal species of *H*. *japonica* were several mammalian species (i.e., sika deer [*Cervus nippon*], wild boar [*Sus scrofa*], Japanese serow [*Capricornis crispus*], Japanese macaque [*Macaca fuscata*], Japanese hare [*Lepus brachyurus*], and Carnivora), and two avian species (*Phasianus soemmerringii* or *P*. *colchicus versicolor*; Yoshiba & Abe, 1989; Nakanojo Town, 2004; Sasaki et al., 2005; Sasaki & Tani, 2008; Kanagawa Prefecture, 2009). Our present study identified four of the abovementioned host animal species, including sika deer, wild boar, Japanese serow, and Japanese hare (Figure 2; Appendix S2). In addition, the present study identified new host animal species, including the raccoon dog (*Nyctereutes procyonoides*), Asian black bear (*Ursus thibetanus*), red fox (*Vulpes vulpes*), Japanese weasel (*Mustela itatsi*), feral cat (*Felis catus*), and Siberian chipmunk (*Tamias sibiricus*; local alien population; Mito & Uesugi, 2004). The identification of Ursidae (bear) and Sciuridae (squirrel) species from iDNA isolated from other land leech species has also been reported in South‐East Asia (Schnell et al., 2018). The result of the present study suggest that *H*. *japonica* also feeds on a wide range of large‐bodied Carnivora species (e.g., Asian black bear) and small mammals (e.g., Japanese weasel and Siberian chipmunk), but perhaps as infrequently as has been previously reported in other Asian *Haemadipsa* species.

Notably, the present study revealed that several *H. japonica* fed on blood meals from frog species (Figure 2; Appendix S2). Though it is known that *H*. *japonica* feeds on frog blood meals in the experimental rearing environments (Yoshiba, 1996), only two previous studies have reported that *H*. *japonica* feeds on frogs and salamander in the wild in Japan. One of the aforementioned studies discovered that iDNA of *H*. *japonica* belonged to Yakushima Tago's brown frog (*Rana tagoi yakushimensis*) from Yakushima Island (Hanya et al., 2019). The other study found that a haemadipsid leech (identified as *H*. *zeylanica japonica*) fed on Sword‐tail newts (*Cynops ensicauda*) in the Ryukyu islands (Miyata, Miyagi, & Tsukamoto, 1978). However, several studies outside of Japan have described frogs as hosts of various land leech species (e.g., Rocha, Borda, Andreone, & Rosa, 2012; Schnell et al., 2018), and salamanders have also been infrequently described as hosts (Lunghi et al., 2018). This lack of studies reporting on amphibians as hosts of *H*. *japonica* can be attributed to the analytical bias that is present when using DNA analyses or immunohistological analyses; amphibians were not used as candidates for host species identification in previous studies using fragment size‐based DNA analyses or immunohistological analyses. In general, amphibians dwell in moist, mountainous habitats, such as along small streams and damp areas. Therefore, the habitats of amphibians overlap with the habitats of *H*. *japonica*, and consequently, the leech has opportunities to feed on blood meals from amphibians.

Recently, host animal species of land leeches have been identified by metabarcoding analyses of pooled (mixed) iDNA from several collected leeches using high throughput sequencing on next generation sequencing (NGS; e.g., Drinkwater et al., 2019; Schnell et al., 2018). These NGS techniques are highly effective at detecting host species than that of Sanger sequencing method for iDNA. On the contrary, the use of NGS techniques on iDNA can present imperfect species identifications resulting from false positives or negative detections (Drinkwater et al., 2019). Our present study (i.e., 17 host species from 144 leeches/826 collected leech; Appendices S1 and S2) may underestimate the number of *H*. *japonica* host species due to lower detection efficacy caused by isolating DNA only from leeches with blood clots and possibly by relatively longer lengths of 16S rDNA PCR fragments (ca. 460 bp) than currently used 16S rDNA PCR fragments (294 bp; Weiskopf et al., 2018). However, the present study provides solid evidence for the host species of *H*. *japonica* with high accuracy of detection and lays the foundation for future studies using NGS techniques.

### 4.2 The host–parasite interface between *H. japonica* and *C. nippon*

The result of NMDS and *k*‐means clustering in this study showed that the host animal species compositions of the sites were divided into two groups, which were mainly separated according to sika deer presence or absence (Figure 3). In addition, the results of the MRPP and ANOSIM analyses suggested that host species composition differed significantly between sites with sika deer and sites without sika deer. These results strongly indicate that sika deer distribution affects the host–parasite interface of *H. japonica*. Since around the 1990s, the populations of sika deer have substantially increased in Japan (Takatsuki, 2009). Therefore, *H*. *japonica* might easily find and attack sika deer as abundant easy prey.

Furthermore, the GLMMs demonstrated that the number of *H*. *japonica* samples that fed on Carnivora and Anura samples decreased in sites where sika deer were present (Table 1), whereas in the sites without sika deer members of the Anura order are significant or nearly significant indicator species (Table 2). These results indicate that Anura species are especially the most important host of *H*. *japonica* in sites where sika deer are not found. Previous laboratory studies have demonstrated that *H*. *japonica* prefers homothermal animals to poikilothermic animals (Yoshiba, 1996). In addition, Davies and McLoughlin (1996) found that mammalian blood provides more energy than amphibian blood, resulting in more available energy for somatic and reproductive growth in an aquatic leech, *Hirudo medicinalis*.

Therefore, the present study suggests that *H*. *japonica* populations may have increased via a change of host preference to sika deer from frog species, as a result of the substantial expansion of sika deer populations and subsequent increase in the frequency that *H*. *japonica* feeds on the sika deer, as well as by the subsequent increases in reproductive energy obtained from sika deer blood meals compared to those of frog species. In summary, the distribution of sika deer results in increased *H*. *japonica* populations through a change in the host–parasite interface.

Notably, for *Hirudo medicinalis*, previous studies have suggested that an opposite change of host preference may have occurred. That is, recent changes in cattle and deer management have decreased their availability to *H*. *medicinalis*, resulting in amphibians becoming the primary hosts and leading to decreased *H*. *medicinalis* populations due to the low reproductive energy obtained from amphibian blood meals (Davies & McLoughlin, 1996; Wilkin & Scofield, 1990). These previous studies and the present study, taken together, imply that management focusing on deceasing sika deer populations is an effective method to reduce *H. japonica* populations. This may open a window for the application of iDNA to forest ecology management.

## CONFLICT OF INTERESTS

None declared.

## AUTHOR CONTRIBUTION

**Kaori Morishima:** Conceptualization (lead); Data curation (lead); Formal analysis (lead); Investigation (lead); Methodology (lead); Visualization (lead); Writing‐original draft (lead); Writing‐review & editing (lead). **Takafumi Nakano:** Conceptualization (supporting); Formal analysis (equal); Funding acquisition (lead); Methodology (supporting); Writing‐original draft (equal). **Mineaki Aizawa:** Conceptualization (equal); Formal analysis (equal); Funding acquisition (lead); Investigation (equal); Methodology (supporting); Project administration (equal); Writing‐original draft (equal); Writing‐review & editing (supporting).

## Supporting information

Appendix S1‐S4

Supplementary Material

## Data Availability

All mitochondrial DNA (mtDNA) sequences from iDNA of *H. japonica* have been deposited in GenBank under accession numbers LC500148–LC500170 and LC500172–LC500200.
