# Supplementary material for: Sika deer presence affects the host–parasite interface of a Japanese land leech
Source: Ecol Evol. 2020 May 19;10(12):6030–8. doi: 10.1002/ece3.6344 (PMC7319141; doi:10.1002/ece3.6344)
Supplement: Supplementary file 2 — Supplementary Material [file ECE3-10-6030-s002.docx]

**Supporting Information**

**Appendix S1** Collection sites and number of *Haemadipsa japonica* samples analysed per site. † indicates the absence of *Cervus nippon*; Lat., Long., and Alt. indicate latitude, longitude, and altitude of the sampling location, respectively; *N*d, *N*i, and *N* indicate the number of dissected leeches, the number of dissected leeches with blood clots from which invertebrate-derived DNA (iDNA) was isolated, and the number of host animal species identified from the leech, respectively. Numbers in parentheses indicate breakdown of pooled samples in each site.

**Appendix S2** Host animal species identified using 16S rRNA mitochondrial DNA sequences and BLAST for invertebrate-derived DNA (iDNA) from bloodmeals of *Haemadipsa japonica*. † indicates the absence of sika deer (*Cervus nippon*); ID (%) indicates per cent identity; *N* indicates the number of *Haemadipsa japonica* samples that fed on the assigned taxa; § was identified using cytochrome b fragments; Collection site numbers (1–26) correspond to those shown in Appendix S1.

**Appendix S3** Host animal species identified using 16S rRNA and cytb mitochondrial DNA sequences and BLAST for invertebrate-derived DNA (iDNA) from bloodmeals of *Haemadipsa japonica* individuals

**Appendix S4** The predicted values (Pred) and 95% confidence intervals (CI; Lower and Upper) for *Haemadipsa japonica* samples that fed on wild boar (*Sus scrofa*) (*N*ss), Japanese serow (*Capricornis crispus*) (*N*cc), carnivores (Carnivora) (*N*ca), and frog species (Anura) (*N*an) per the site, which were obtained using generalised liner mixed models (GLMMs). ‡ indicates the predicted value ± square root of the sum of the squares of the differences between the lower and upper limits of 95% CI and predicted values in each site.
